# Complete chloroplast genome of Ulleung Island endemic flowering cherry, *Prunus takesimensis* (Rosaceae), in Korea

**DOI:** 10.1080/23802359.2018.1443034

**Published:** 2018-02-24

**Authors:** Myong-Suk Cho, Ji Young Yang, Seung-Chul Kim

**Affiliations:** a Department of Biological Sciences, Sungkyunkwan University, Suwon, Republic of Korea;; b Research Institute for Ulleung-do & Dok-do, Kyungpook National University, Daegu, Republic of Korea

**Keywords:** Chloroplast, endemic flowering cherry, Ulleung Island, *Prunus takesimensis*

## Abstract

*Prunus takesimensis* is an endemic flowering cherry on Ulleung Island, Korea. The complete chloroplast (cp) genome of *P. takesimensis* was generated by *de novo* assembly using whole-genome next-generation sequencing approach. The cp genome size was 157,948 bp in length consisting of four regions; large single copy region (85,959 bp), small single copy region (19,117 bp), and a pair of inverted repeat regions (26,436 bp). The genome contained a total of 128 genes, including 83 coding genes, 8 rRNA genes, and 37 tRNA genes. Phylogenetic analysis for 20 reported genomes within the family Rosaceae showed the monophyly of the genus *Prunus* including newly sequenced *P. takesimensis.*

The wild flowering cherry, *Prunus takesimensis* Nakai is distributed on Ulleung Island, Korea. Morphologically, *P. takesimensis* is the most similar to *P*. *sargentii*, which is found in the adjacent continent and islands (i.e. Jeju Island) of Korea, Japanese Archipelago, and Russian Far East (Ohwi 1965; Kim 2009). Due to the morphological similarities shared by both species, *P*. *sargentii* is presumed as the continental progenitor of *P. takesimensis*. The Ulleung Island endemic *P. takesimensis* has been recognized from its sister species primarily based on reproductive traits: smaller flowers (26–32 mm in diameter) and more flowered (3–5) inflorescence (Chang et al. 2004). However, the genetic relationship of *P. takesimensis* relative to other flowering cherries has not been well established in the previous phylogenetic analyses using several nuclear and chloroplast markers (Cho 2017). Therefore, we sequenced the whole chloroplast genome of *P. takesimensis* to elucidate its evolutionary history on Ulleung Island in the context of the phylogenetic relationship among other flowering cherries.

Total genomic DNA was isolated from fresh leaves sampled from natural habitat (37°29′3.83″N, 130°54′44.37″E, Alt. 132m) in Ulleung Island using the DNeasy Plant Mini Kit (Qiagen, Carlsbad, CA). Voucher specimen (*SKK* *Cho et al.* *PT1)* was deposited in the herbarium of Sungkyunkwan University (SKK). Paired-end genomic library was constructed and sequenced using Illumina HiSeq 4000 platform. Total reads of 10.95 Gb (Order 1611AHF-0002/sample PT1) were obtained and assembled by *de novo* genomic assembler, Velvet 1.2.10 (Zerbino and Birney 2008). The representative chloroplast contig (>521×) was retrieved and aligned against the reference chloroplast genome of *P. serrulata* var. *spontanea* (GenBank accession KP760073) using Geneious v8.1.6 (Biomatters Ltd., Auckland, New Zealand). Annotation was performed using Dual Organellar GenoMe Annotator (Wyman et al. 2004), ARAGORN v1.2.36 (Laslett and Canback 2004) and RNAmmer 1.2 Server (Lagesen et al. 2007).

The complete chloroplast genome of *P. takesimensis* (GenBank accession MG754959) was 157,948 bp in length consisting of four regions; large single copy region (LSC) of 85,959 bp, small single copy region (SSC) of 19,117 bp, and a pair of inverted repeat regions (IRA and IRB) of each 26,436 bp. The overall GC contents of the chloroplast genome were 36.7%; LSC (34.6%), SSC (30.2%), and IR (42.5%) regions. The genome contained a total of 128 genes, including 83 coding genes, 8 rRNA genes, and 37 tRNA genes.

Phylogenetic analysis was conducted to confirm the phylogenetic position of newly sequenced *P. takesimensis* amongst 20 representative species of Rosaceae. The complete chloroplast genome sequences were aligned using MAFFT v.7 (Katoh and Standley 2013) to produce the maximum likelihood (ML) tree by IQ-TREE (Nguyen et al. 2014) with 1000 replicate bootstrap (BS) analyses. The ML tree (Figure 1) showed that the genus *Prunus* was strongly supported as monophyletic group (BS 100%) and *P. takesimensis* was most closely related to *P. serrulata* var. *spontanea*.

**Figure 1.**
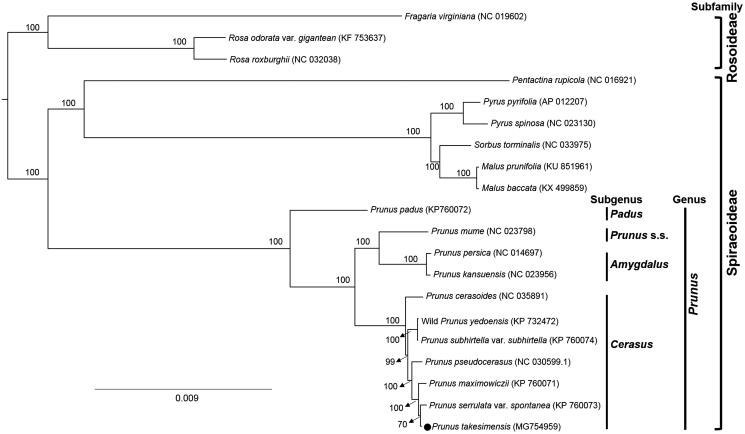
Maximum likelihood tree of the Rosaceae species constructed by IQ-TREE. Twenty representative complete chloroplast genome sequences were used in the analysis including *P. takesimensis* (GenBank accession MG754959). The percentage of bootstrap support value calculated with 1000 bootstrap replications is shown on each node. The phylogenetic tree is drawn to scale, with branch lengths measured in the number of substitutions per site.
